# Chronic Disease Counseling and Screening by Dental Professionals: Results From NHANES, 2011–2016

**DOI:** 10.5888/pcd17.200152

**Published:** 2020-08-20

**Authors:** Eleanor Fleming, Astha Singhal

**Affiliations:** 1 National Center for Health Statistics, Centers for Disease Control and Prevention, Hyattsville, Maryland; 2 Health Policy and Health Services Research, Henry M. Goldman School of Dental Medicine, Boston University, Boston, Massachusetts

## Abstract

**Introduction:**

Dental visits may provide an opportunity to counsel and screen for chronic disease prevention. However, few studies have used nationally representative data to assess the potential role of dental professionals in chronic disease prevention. We examined the percentage of US adults who reported chronic disease counseling and screening by dental professionals.

**Methods:**

We analyzed data from the National Health and Nutrition Examination Survey 2011–2016 for 5,541 participants aged 30 or older who reported seeing a dental professional in the past year and estimated the percentage who reported receiving counseling about selected chronic disease prevention during the visit. We used logistic regressions to examine associations between risk factors and counseling.

**Results:**

Overall, 4.0% (standard error [SE], 0.3) of adults were told by a dental professional about the benefits of checking blood glucose, 42.4% (SE, 2.9) giving up tobacco (among tobacco users), 26.6% (SE, 1.2) about checking for oral cancer, and 43.0% (SE, 1.8) had an oral cancer examination. Groups with risk factors were more likely to receive health behavior counseling than those without (eg, those previously told they had diabetes risk factors were more likely to receive blood glucose counseling than those without [8.1% vs 3.3%, *P* < .05]). The pattern for oral cancer counseling and receiving an oral cancer examination was different: adults without oral cancer risk factors (no tobacco use, normal/underweight, and/or excellent/very good health) were more likely to receive oral cancer counseling or screening. Adjusted analyses did not change these associations.

**Conclusion:**

Most adults were not counseled about chronic disease prevention during a visit with a dental professional. Current tobacco users and those with overweight or obesity were more likely to report receiving counseling.

**Summary**

**What is already known about this topic?**

Each year approximately 27 million people visit a dentist but not a physician. Dental care settings provide an opportunity for chronic disease screening and health promotion.

**What is added by this report?**

We used data from the National Health and Nutrition Examination Survey 2011–2016 to estimate the percentage of US adults who were advised about chronic disease prevention by a dental professional in the past year.

**What are the implications for public health practice?**

Most adults were not advised about chronic disease prevention during a visit with a dental professional. Current tobacco users and those with overweight or obesity were more likely to report receiving counseling.

## Introduction

Chronic disease prevention and early diagnosis are important for population health and can greatly improve health outcomes (1,2). In the United States, 6 in 10 adults have a chronic disease, and 4 in 10 have 2 or more chronic diseases (2). Cancer and diabetes remain among the leading causes of disease and death in the United States (3,4). According to the Centers for Disease Control and Prevention, 34.1 million adults aged 18 or older (13.0% of the 2018 US population) had diabetes in 2018, and 88 million had prediabetes (34.5% of the adult US population) (5). In 2016, the most recently available data, 45,543 new diagnoses of oral cavity and pharynx cancer were reported in the United States, and 10,170 people died of those diseases (6). Diabetes and oral cancer have known risk factors. For example, people who are overweight are at risk for developing prediabetes and diabetes (7). Smoking also increases the risk of developing diabetes (8). Tobacco use is a risk factor for oral cancer (9,10).

Each year approximately 27 million people visit a dentist but not a physician (10). Dental care settings provide an opportunity for chronic disease screenings and health promotion (11,12). Screening for chronic disease in dental offices could reduce US health care costs by up to $102.6 million per year (12). Although previous studies focused on chronic disease screenings in dental settings to assess cost savings, few studies used nationally representative data (13). We used the most recently available nationally representative data to assess the extent to which dental professionals screened and counseled patients for chronic disease prevention and to determine the factors associated with receiving counseling. We hypothesized that such counseling from dental professionals was higher among patients with identified risk factors for chronic disease than those without.

## Methods

### Study sample

We estimated the percentage of US adults who were advised about chronic disease prevention during a visit with a dental professional in the past year. We used National Health and Nutrition Examination Survey (NHANES) data to assess differences by demographic characteristics and chronic disease risk factors, including current tobacco use and body weight. Three NHANES survey cycles (2011–2012, 2013–2014, and 2015–2016) were combined to provide stable estimates. NHANES is a cross-sectional survey administered by the National Center for Health Statistics (NCHS) to monitor the health and nutritional status of the civilian, noninstitutionalized US population. NHANES uses a highly stratified, multistage probability sampling design (14). Since 1999, NHANES has been collected continuously and data released in 2-year cycles. The survey consists of interviews conducted in participants’ homes and standardized health examinations and biospecimen collection conducted in mobile examination centers (15). All adult participants provide written informed consent. In NHANES 2011–2016, non-Hispanic black, non-Hispanic Asian, and Hispanic participants, in addition to other groups, were oversampled to obtain reliable estimates for these population subgroups (16). The examination response rate for all NHANES respondents ranged from 69.5% in 2011–2012 to 68.5% in 2013–2014 and 58.7% in 2015–2016 (14). The NCHS Research Ethics Review Board approved the NHANES protocol.

Adults aged 30 and older who had a dental visit in the past 12 months were eligible for our study if they reported in response to the oral health questionnaire that a dental professional advised them about chronic disease risk factors and counseled or screened them in all of the following 4 areas: benefits of giving up cigarettes and other types of tobacco, benefits of checking blood glucose, importance of checking for cancer, and receiving oral cancer exam in which the doctor pulled on the tongue. Those missing any of the 4 were excluded from the sample (15). Although respondents younger than 30 completed the oral health questionnaire, they were not asked about oral cancer screenings and were thus not eligible for inclusion in our study. To each of these questions, respondents could answer: yes, no, refused, or did not know. For the analysis, only yes and no responses were used. Of 7,745 possible participants, 13 had missing or “do not know” responses for being told the benefits of checking blood glucose (0.2%), 9 for being told the benefits of giving up tobacco (0.1%), 22 for being told importance of checking for oral cancer (0.3%), and 52 for having an oral cancer examination (0.7%). After excluding these participants with missing outcome data, 7,649 NHANES participants were eligible and included in our study.

Chronic disease risk factors were defined by using questionnaire and examination data. Key risk factors were current tobacco use, being previously told about having an increased diabetes risk, having overweight or obesity, fair or poor self-assessed general health status, and fair or poor self-assessed oral health status. Current tobacco users were identified on the basis of responses to questions about tobacco use in the past 5 days. If respondents replied yes to using cigarettes, cigars, smokeless tobacco, chewing tobacco, or snuff in the past 5 days, they were characterized as current tobacco users. If respondents replied no, they were characterized as not being current tobacco users. E-cigarettes were included only in the 2013–2014 survey cycle so were not included in our analysis. Respondents were asked if they were told by a doctor or other health professional that they were at risk for developing diabetes because of a health condition or family or personal medical history. If respondents answered yes, they were characterized as told previously they had diabetes risk, and if no, were characterized as not being told previously they had diabetes risk.

Body mass index (BMI) was calculated by using height and weight measured by using standardized procedures as weight in kilograms divided by height in meters squared (kg/m2). BMI in the range of 25 to <30 is defined as overweight, and BMI that is 30 or higher is defined as obesity. For this study, we categorized weight status as normal/underweight (BMI <25) versus overweight/obesity (BMI ≥25). We grouped overweight and obesity together because BMI in this range increases risk for diabetes and other chronic diseases (17). Self-assessed health status is a measure of how a person perceives his or her health and is related to a person’s perception of risk of disease (18,19). We based self-assessed health status on participants describing their health as excellent, very good, good, fair, or poor. For our analysis, we categorized general health status as excellent/very good, good, and fair/poor. Self-reported oral health status was based on the question that asked study participants to rate the health of their teeth and gums as excellent, very good, good, fair, or poor. We categorized oral health status as excellent/very good, good, and fair/poor.

We selected the following covariates from the questionnaire’s demographic data files on the basis of their previous analyses and availability in NHANES: age, sex, race/ethnicity, poverty status, and education (5,9). Age was categorized as 30 to 44, 45 to 64, 65 to 74, and 75 or older. Sex was categorized as male or female. Race/ethnicity was categorized as non-Hispanic white, non-Hispanic black, non-Hispanic Asian, Hispanic, and other. Hispanic origin included Mexican American and other Hispanic ethnicities. Race/ethnicity estimates reflect people reporting only 1 race. Poverty status was based on family income and family size according to the US Census Bureau’s poverty thresholds for the previous calendar year. Poverty status was defined as a family income less than 200% of the federal poverty level (FPL) or greater than or equal to 200% FPL. Education was categorized as less than a high school diploma, high school diploma or general equivalency diploma (GED), or more than a high school diploma or GED (some college, associate degree, or college degree or above).

We excluded participants from our analysis with missing responses for risk factors and other covariates (n = 2,108, 27.6%) for a final analytic sample of 5,541. Covariates with more than 5% missing responses were current tobacco use status (n = 1,051, 13.4%) and risk of diabetes (n = 999, 15.2%). Because of missing covariate data, participants included were more likely to be younger than those excluded, and more were non-Hispanic white. To assess if excluding observations with missing data could lead to bias, we used PROC WTADJUST in SUDAAN (RTI International) to reweight the data to achieve population totals for age group and race/ethnicity before exclusions. We compared study outcomes obtained with the recalculated weights to those obtained with the original exam weights. Estimates for the prevalence of receiving counseling did not vary more than 10 percent, and we saw no differences in direction and significance of association. Therefore, we used the original survey weights in the analyses.

### Statistical methods

We estimated percentages (and standard errors) of adults who reported being told about the benefits of chronic disease prevention by tobacco use status, weight, sex, age group, race/ethnicity, income, and education. Associations between the 4 chronic disease prevention questions and each covariate were tested using the Satterthwaite-adjusted χ^2^ test with Rao-Scott correction at the *P* < .05 significance level. We used pairwise comparisons to evaluate differences between specific categories. Because being told the benefits of giving up tobacco was only applicable to current tobacco users, the analyses for tobacco use counseling included only current tobacco users (n = 843). Multiple logistic regression analysis was used to evaluate the association between chronic disease risk factors and covariates. The multiple logistic regression model included all covariates: demographic characteristics, current tobacco use, weight status, diabetes risk, general health status, and oral health status. We calculated adjusted odds ratios and 95% confidence intervals.

To account for the differential probabilities of selection, nonresponse, and noncoverage, sample weights from the NHANES mobile examination center were incorporated into the estimation process. In estimating standard errors, the complex sample design was incorporated by using Taylor series linearization with provided survey design variables (20). Adjustments were not made for multiple comparisons. We used SAS System for Windows, release 9.3 (SAS Institute Inc) and SUDAAN, release 11.1 (RTI International) to conduct statistical analyses.

## Results

Among our sample of 5,541 respondents, 14.8% were current tobacco users, 71.0% had overweight/obesity, 14.1% were told previously that they had diabetes risk factors, 11.0% reported fair/poor general health, and 17.0% reported fair or poor oral health (Table 1). Overall, 54.5% (SE, 1.6) of adults who visited a dental professional in the past year reported receiving any of the 4 specific chronic disease counseling messages or screening exam.

**Table 1:** Characteristics of US Adults (N = 5,541) Aged ≥30 Who Visited a Dental Professional in the Past 12 Months, NHANES, 2011–2016^a^

Characteristic	Sample Distribution^b^	
	No.	Weighted Percentage (SE)
**Age, y**		
30–44	1,801	31.3 (1.2)
45–64	2,301	45.1 (1.1)
65–74	832	14.9 (0.8)
≥75	607	8.7 (0.6)
**Sex**		
Male	2,464	44.8 (0.8)
Female	3,077	55.2 (0.8)
**Race/ethnicity**		
White, non-Hispanic	2,463	74.7 (1.7)
Black, non-Hispanic	1,080	8.5 (0.9)
Asian, non-Hispanic	712	4.8 (0.6)
Hispanic	1,128	9.5 (1.0)
Other	158	2.5 (0.3)
**Poverty status, % of federal poverty level**		
<200%	1,749	21.7 (1.4)
≥200%	3,372	78.3 (1.4)
**Education**		
<High school	839	9.1 (0.9)
High school graduate	1,050	17.4 (0.9)
>High school	3,652	73.5 (1.4)
**Current tobacco use**		
Yes	873	14.8 (0.8)
No	4,668	85.2 (0.8)
**Body mass index (weight in kg/height in m^2^) **		
Overweight/obese (≥25)	3,898	71.0 (0.9)
Normal weight/underweight (<25)	1,643	29.0 (0.9)
**Previously told had diabetes risk**		
Yes	844	14.1 (0.5)
No	4,697	85.9 (0.5)
**General health**		
Excellent/very good	2,416	51.5 (1.3)
Good	2,241	37.5 (1.0)
Fair/poor	884	11.0 (0.7)
**Oral health**		
Excellent/very good	2,279	49.7 (1.4)
Good	1,990	33.3 (1.0)
Fair/poor	1,272	17.0 (0.9)

Abbreviation: NHANES, National Health and Nutrition Examination Survey; SE, standard error.

a Sample included adults who reported that the dental professional advised them about chronic disease risk factors and counseled or screened them in all of the following 4 areas: benefits of giving up cigarettes and other types of tobacco, benefits of checking blood glucose, importance of checking for cancer, and receiving oral cancer exam in which the doctor pulled on the tongue. Those missing any of the 4 were excluded from the sample.

b Percentages are weighted to be nationally representative.

Only 4.0% (SE, 0.3) of adults 30 years or older who had a dental visit in the past 12 months reported that they were ever told by a dental professional about the benefits of checking their blood glucose (Table 2). We found differences in their being told about the benefits of checking blood glucose for all risk factors. A higher percentage of adults who used tobacco, had overweight/obesity, and were previously told they had diabetes risk factors received blood glucose counseling than adults without these risk factors. A higher percentage of adults who reported fair/poor oral health status received blood glucose counseling than those who reported good or excellent/very good oral health (*P* < .05).

**Table 2:** Percentage of US Adults Aged ≥30 (N = 5,541) Reporting Chronic Disease Counseling and Screening During a Visit to a Dental Professional in the Past 12 Months, by Risk Factor Status, NHANES 2011–2016^a^

Characteristic	Told Benefits of Checking Blood Glucose (n = 5,541)	Told Benefits of Giving up Tobacco^b^ (n = 873)	Told Importance of Checking for Oral Cancer (n = 5,541)	Had Oral Cancer Examination (n = 5,541)
**Overall**	4.0 (0.3)	42.4 (2.9)	26.6 (1.2)	43.0 (1.8)
Yes	8.1 (1.2)^c^	42.4 (2.9)	26.3 (2.5)	30.7 (2.8)^c^
No	3.3 (0.4)^c^	Not applicable	26.7 (1.2)	45.1 (1.9)^c^
**Body mass index (weight in kg/height in m^2^)**				
Overweight/obesity (>25)	4.3 (0.5)	42.9 (3.0)	27.0 (1.3)	41.0 (2.0)^c^
Normal/underweight (≤25)	3.3 (0.5)	41.1 (4.4)	25.7 (1.6)	47.6 (2.1)^c^
**Previously told had diabetes risk**				
Yes	8.1 (1.3)^c^	47.7 (5.5)	31.4 (2.2)^c^	45.2 (3.0)
No	3.3 (0.4)^c^	41.3 (3.0)	25.8 (1.3)^c^	42.6 (1.9)
**General health**				
Excellent/very good	3.7 (0.6)	40.0 (4.3)	28.0 (1.7)	50.7 (2.1)^c^
Good	3.9 (0.6)	44.5 (3.9)	26.3 (1.8)	38.1 (2.1)^c^
Fair/poor	5.9 (1.0)	41.9 (5.1)	21.4 (2.6)	22.9 (2.2)^c^
**Oral health**				
Excellent/very good	3.5 (0.6)^c^	32.1 (4.2)^c^	29.7 (1.4)^c^	52.6 (2.0)^c^
Good	3.7 (0.6)^c^	43.5 (4.1)^c^	24.7 (1.9)^c^	38.5 (2.1)^c^
Fair/poor	5.9 (0.9)^c^	51.1 (4.5)^c^	21.4 (1.7)^c^	23.6 (2.1)^c^

Abbreviation: NHANES, National Health and Nutrition Examination Survey.

a Values are weighted percentage (standard error). Percentages are weighted to be nationally representative.

b Includes current tobacco users only.

c Test of significance based on stratum-adjusted Cochran-Mantel-Haenszel χ^2^ tests of general association at the *P* < .05 significance level.

Overall, 42.4% (SE, 2.9) of tobacco users who visited a dental professional in the past year reported being told about the benefits of giving up tobacco. A higher percentages of adults who used tobacco and who reported fair/poor oral health status were told the benefits of giving up tobacco compared with tobacco users who reported good or excellent/very good oral health (*P* < .05).

Among the total study group, 26.6% (SE, 1.2) reported being told about the importance of checking for oral cancer. Although tobacco use is a risk factor for oral cancer, similar percentages of current tobacco users (26.3%) and nonusers (26.6%) reported receiving this counseling. However, higher percentages of adults with overweight/obesity and those with previously identified diabetes risk factors were told about the importance of checking for oral cancer than those without these risk factors. People who reported excellent/very good general and oral health were more likely to be told the importance of checking for oral cancer than those with good or fair/poor general and oral health (*P* < .05). This was the opposite of the pattern for checking blood glucose and giving up tobacco where those in fair/poor health were more likely to receive counseling.

Among people aged 30 years or older, 43.0% (SE, 1.8) reported having an oral cancer examination. Although tobacco use is a risk factor for oral cancer, a lower percentage of current tobacco users (30.7%) reported receiving an oral exam than nonusers (45.1%). We found differences by all risk factors except for diabetes risk. By general and oral health status, we found significant pairwise comparisons by all pairs (*P* < .05). People who reported no current tobacco use and excellent/very good general and oral health and whose weight status was normal/underweight were more likely to receive an oral cancer examination, again the opposite pattern for these risk factors compared with counseling for blood glucose and tobacco use where people at risk were more likely to receive counseling.

Adjusted odds ratios (AORs) of receiving chronic disease counseling and screening by a dental professional were similar to the crude odds ratio (ie, none changed more than 10% with adjustment). Thus, only adjusted results are presented (Table 3). Current tobacco users (AOR = 2.20; 95% CI, 1.53–3.17) and adults told previously they had diabetes risk (AOR = 2.33; 95% CI, 1.64–3.32) had higher odds of being told the benefits of checking blood glucose. Adults with fair/poor oral health status (AOR = 2.41; 95% CI, 1.41–4.10) had the highest odds of being told the importance of giving up tobacco. Current tobacco users (AOR = 1.33; 95% CI, 1.02–1.74) and adults told previously they had diabetes risk (AOR = 1.37; 95% CI, 1.10–1.71) had greater odds of being told the importance of checking for oral cancer than adults who did not currently use tobacco and who had not been told previously they had diabetes risk.

**Table 3:** Adjusted Odds of Being Counseled About or Screened for Chronic Disease During a Dentist Visit in the Past 12 Months, Adults Aged ≥30 (N = 5,541), NHANES 2011–2016^a^

Chronic Disease Risk Factor	Told Benefits of Checking Blood Glucose (n = 5,541)	Told Benefits of Giving up Tobacco^b^ (n = 873)	Told Importance of Checking for Oral Cancer (n = 5,541)	Had Oral Cancer Examination (n = 5,541)
**Currently use tobacco**				
Yes	2.20 (1.53–3.17)	Not applicable	1.33 (1.02–1.74)	0.96 (0.70–1.31)
No	Reference	Not applicable	Reference	Reference
**Body mass index (weight in kilograms/height in meters^2^)**				
Overweight/obesity (≥25)	1.12 (0.77–1.64)	1.09 (0.72–1.65)	1.07 (0.90–1.28)	0.82 (0.68–0.99)
Normal/underweight (<25)	Reference	Reference	Reference	Reference
**Previously told had diabetes risk**				
Yes	2.33 (1.64–3.32)	1.24 (0.74–2.10)	1.37 (1.10–1.71)	1.41 (1.07–1.84)
No	Reference			
**General health**				
Excellent/very good	Reference			
Good	0.78 (0.53–1.16)	1.08 (0.73–1.60)	1.05 (0.84–1.32)	0.91 (0.75–1.11)
Fair/poor	0.75 (0.46–1.24)	0.82 (0.45–1.51)	0.89 (0.61–1.29)	0.63 (0.46–0.86)
**Oral health**				
Excellent/very good	Reference			
Good	0.88 (0.64–1.20)	1.65 (0.96–2.83)	0.82 (0.67–1.00)	0.74 (0.61–0.89)
Fair/poor	1.08 (0.69–1.68)	2.41 (1.41–4.10)	0.78 (0.60–0.99)	0.51 (0.41–0.63)

a Values are adjusted odds ratios (95% confidence intervals). Ratios were adjusted for chronic disease risk factors (overweight/obesity, diabetes risk, tobacco use) and for sex, age, race/ethnicity, poverty status, and education. Data source: National Health and Nutrition Examination Survey, 2011–2016 (16).

b Includes only current tobacco users.

Adults who reported fair/poor oral health status had lower odds of being told the importance of checking for oral cancer (AOR= 0.78; 95% CI, 0.60–0.99) than those with excellent or very good oral health status. We found no association between current tobacco use and being counseled about checking for oral cancer. Adults with overweight/obesity weight status had lower odds of having an oral cancer exam, compared with adults with normal/underweight weight status (AOR = 0.82; 95% CI, 0.68–0.99). Adults told previously that they had an increased risk for diabetes had greater odds of having an oral health examination (AOR = 1.41; 95% CI, 1.07–1.84) than adults not told previously. Adults with fair/poor general health status had lower odds of having an oral cancer examination (AOR = 0.63; 95% CI, 0.46–0.86) than adults with excellent or very good general health status. Adults with good oral health status (AOR = 0.74; 95% CI, 0.61–0.89) and fair/poor oral health status (AOR = 0.51; 95% CI, 0.41–0.63) had lower odds of having an oral cancer examination than adults with self-reported health status of excellent/very good.

## Discussion 

Slightly more than 50% of adults who visited a dental professional in the past year reported receiving some counseling about chronic disease prevention or oral cancer screening; however, some risk factors (current tobacco status and weight status) make counseling more likely. Dental professionals typically review a patient’s medical history on the patient intake form before providing treatment, and may use risk factors to target counseling (21). Although NHANES does not provide data on whether dental professionals performed screening and ascertained risk factors, our results suggest that dental care providers were aware of risk factors, either by observation or through patient history (eg, tobacco use, weight). Adults with these risk factors were generally more likely to get counseling for chronic disease prevention than those without the risk factors. However, even among the groups with indications for preventive health counseling, the percentage receiving counseling was still below 50% and sometimes far below.

The results from our study also show that adults not currently using tobacco and adults in excellent and very good general and oral health were more likely to receive oral cancer counseling than those with poor general and oral health. This pattern suggests that the type of counseling adults receive may be viewed differently by dental professionals, that dental professionals may perceive certain patients as being more receptive to counseling, or that visit content may be different for patients in poor health. In the first nationwide survey of practicing general dentists about their attitudes toward chairside screenings for medical conditions, most dentists thought screening was important and were willing to conduct chairside screenings, collect oral fluid samples and blood samples via a finger stick, and refer patients for medical consultations (22). In a nationally representative study of dentists’ willingness to screen for cardiovascular disease in dental care settings, 91.7% of 1,802 dentists who completed the study’s surveys reported that the health history that they used in their clinical practices included questions about tobacco use; 40.0% of responding dentists reported that the health history included questions about obesity, and 99.5% reported that the health history included questions about diabetes (23). It is unclear whether a willingness to screen, refer, and test translates into clinical practice.

Dental professionals suggest that there are barriers to offering tobacco cessation and other counseling services. These include patient resistance, lack of insurance reimbursement, lack of time, and not knowing where to refer the patient (24). However, dental students are being taught to provide health education and perform screenings, and dental students in several studies reported they were receptive to providing these screenings in their future clinical practices (25–27). Additionally, the dental students in these studies agreed generally that tobacco cessation and glucose monitoring were within the scope and responsibility of the dental profession (25,26). Dental students were more likely to support glucose screenings for patients diagnosed with diabetes, which suggests that they might overlook patients undiagnosed but at risk for diabetes (27).

In a randomized trial, current smokers in public dental clinics were randomly assigned to receive usual care or an intervention in which the dental professional provided advice and brief counseling on tobacco cessation (28). Participants in the intervention group had higher tobacco use abstinence rates at the 7.5-month follow-up and prolonged abstinence, suggesting that tobacco cessation services provided in dental care settings may help people to quit. That study suggested that dental professionals may improve the overall health of their patients by performing these screenings and providing chronic disease prevention information (28). Moreover, studies have shown that screenings in dental care settings are effective and that patients and providers are willing to participate in these screenings (22–28).

Our study focused on the risk factors associated with receiving chronic disease counseling. We did not analyze how well dental professionals identified patients at risk. Knowing if dental professionals asked about current tobacco use and weight status might help to understand how well dental professionals identified patients at risk. For dental professionals to reach patients with risk factors for chronic disease, they first would need to identify patients at risk. The results from our study suggest indirectly that dental professionals may be aware of their patients’ risk status.

Our study has limitations. First, the data are self-reported and subject to recall and social desirability bias. Because patients have dental visits to have their teeth examined and cleaned or for dental procedures, they may not recall being asked about chronic disease prevention. The self-reported data are also subject to social desirability bias. NHANES does not collect any data directly from dental professionals; thus, actual content of dental visits was not available. Additionally, our analysis focused on study participants who saw a dental professional in the past year but did not include information about their last visit to a medical care provider. This is an important question to consider; however, our study focused solely on chronic disease counseling and screening provided in a dental setting.

Although most US adults aged 30 or older who saw a dental professional in the past year reported receiving routine disease prevention information during their visit, less than 50% received each specific type of counseling. In the past 12 months 4.0% were told about checking blood glucose, 42.4% of tobacco users were told about the benefits of giving up tobacco, 26.6% were told about checking for oral cancer, and 43.0% received an oral cancer examination. Our results may inform opportunities for the role of dental professionals in promoting chronic disease prevention, especially among patients with key risk factors.

## References

[1] Bernell S , Howard SW . Use your words carefully: what is a chronic disease? Front Public Health 2016;4:159. 10.3389/fpubh.2016.00159 27532034 PMC4969287

[2] Buttorff C , Ruder T , Bauman M . Multiple chronic conditions in the United States. Santa Monica (CA): RAND Corporation; 2017. https://www.rand.org/pubs/tools/TL221.html. Accessed June 19, 2020.

[3] Johnson NB , Hayes LD , Brown K , Hoo EC , Ethier KA ; Centers for Disease Control and Prevention (CDC). CDC national health report: leading causes of morbidity and mortality and associated behavioral risk and protective factors — United States, 2005–2013. MMWR Suppl 2014;63(4):3–27. 25356673

[4] Centers for Disease Control and Prevention, National Center for Health Statistics. National vital statistics reports. Vital statistics of the United States, 1980. Volume II — Mortality, part A. 1985; public-use 2016 Mortality File; Xu JQ, Murphy SL, Kochanek KD, Bastian B, Arias E. Deaths: Final data for 2016. Appendix I, National Vital Statistics System. https://www.cdc.gov/nchs/products/nvsr.htm. Accessed June 3, 2020.

[5] Centers for Disease Control and Prevention. National Diabetes Statistics Report, 2017. Atlanta (GA): Centers for Disease Control and Prevention, US Department of Health and Human Services; 2017.

[6] US Cancer Statistics Working Group. United States cancer statistics data visualizations tool, based on November 2018 submission data (1999–2016): US Department of Health and Human Services, Centers for Disease Control and Prevention and National Cancer Institute; www.cdc.gov/cancer/dataviz, June 2019. Accessed June 3, 2020.

[7] Nguyen NT , Nguyen XM , Lane J , Wang P . Relationship between obesity and diabetes in a US adult population: findings from the National Health and Nutrition Examination Survey, 1999–2006. Obes Surg 2011;21(3):351–5. 10.1007/s11695-010-0335-4 21128002 PMC3040808

[8] Blot WJ , McLaughlin JK , Winn DM , Austin DF , Greenberg RS , Preston-Martin S , Smoking and drinking in relation to oral and pharyngeal cancer. Cancer Res 1988;48(11):3282–7. 3365707

[9] Vigneswaran N , Williams MD . Epidemiologic trends in head and neck cancer and aids in diagnosis. Oral Maxillofac Surg Clin North Am 2014;26(2):123–41. 10.1016/j.coms.2014.01.001 24794262 PMC4040236

[10] Vujicic M , Israelson H , Antoon J , Kiesling R , Paumier T , Zust M . A profession in transition. J Am Dent Assoc 2014;145(2):118–21. 10.14219/jada.2013.40 24487594

[11] Genco RJ , Schifferle RE , Dunford RG , Falkner KL , Hsu WC , Balukjian J . Screening for diabetes mellitus in dental practices: a field trial. J Am Dent Assoc 2014;145(1):57–64. 10.14219/jada.2013.7 24379330

[12] Nasseh K , Greenberg B , Vujicic M , Glick M . The effect of chairside chronic disease screenings by oral health professionals on health care costs. Am J Public Health 2014;104(4):744–50. 10.2105/AJPH.2013.301644 24524531 PMC4025684

[13] Strauss SM , Russell S , Wheeler A , Norman R , Borrell LN , Rindskopf D . The dental office visit as a potential opportunity for diabetes screening: an analysis using NHANES 2003–2004 data. J Public Health Dent 2010;70(2):156–62. 10.1111/j.1752-7325.2009.00157.x 20002875

[14] Johnson CL , Dohrmann SM , Burt VL , Mohadjer LK . National health and nutrition examination survey: sample design, 2011-2014. Vital Health Stat 2 2014;(162):1–33. 25569458

[15] Chen TC , Clark J , Riddles MK , Mohadjer LK , Fakhouri THI . National Health and Nutrition Examination Survey, 2015–2018: sample design and estimation procedures. National Center for Health Statistics. Vital Health Stat 2 2018;2(184). 33663649

[16] National Center for Health Statistics. National Health and Nutrition Examination Survey: questionnaires, datasets, and related documentation. https://wwwn.cdc.gov/ nchs/nhanes/Default.aspx. Accessed June 3, 2020.

[17] US Department of Health and Human Services. The Surgeon General’s call to action to prevent and decrease overweight and obesity. Rockville (MD): US Department of Health and Human Services, Public Health Service, Office of the Surgeon General; 2001. 20669513

[18] Idler E , Benyamini Y . Self-rated health and mortality: a review of twenty-seven community studies. J Health Soc Behav 1997;38(1):21–37. 10.2307/2955359 9097506

[19] Lee SJ , Moody-Ayers SY , Landefeld CS , Walter LC , Lindquist K , Segal MR , The relationship between self-rated health and mortality in older black and white Americans. J Am Geriatr Soc 2007;55(10):1624–9. 10.1111/j.1532-5415.2007.01360.x 17697102

[20] Wolter KM . Taylor series methods. In: Wolter KM. Introduction to variance estimation, 2nd edition, p 236–71. New York (NY): Springer Science+Business Media; 2007.

[21] Pickett FA , Gurenlian JR . Preventing medical emergencies: use of the medical history. 2nd edition. Philadelphia (PA): Lippincott, Williams and Wilkins; 2010.

[22] Greenberg BL , Glick M , Frantsve-Hawley J , Kantor ML . Dentists’ attitudes toward chairside screening for medical conditions. J Am Dent Assoc 2010;141(1):52–62. 10.14219/jada.archive.2010.0021 20045822

[23] Singer RH , Feaster DJ , Stoutenberg M , Hlaing WM , Pereyra M , Abel S , Dentists’ willingness to screen for cardiovascular disease in the dental care setting: findings from a nationally representative survey. Community Dent Oral Epidemiol 2019;47(4):299–308; Epub ahead of print. 10.1111/cdoe.12457 30908721 PMC6625893

[24] Prakash P , Belek MG , Grimes B , Silverstein S , Meckstroth R , Heckman B , Dentists’ attitudes, behaviors, and barriers related to tobacco-use cessation in the dental setting. J Public Health Dent 2013;73(2):94–102. 10.1111/j.1752-7325.2012.00347.x 22731618 PMC4028076

[25] Anders PL , Davis EL , McCall WD Jr . Dental students’ attitudes toward tobacco cessation counseling. J Dent Educ 2014;78(1):56–63. 10.1002/j.0022-0337.2014.78.1.tb05657.x 24385525

[26] Anders PL , Davis EL , McCall WD Jr . Dental students’ attitudes toward diabetes counseling, monitoring, and screening. J Dent Educ 2014;78(5):763–9. 10.1002/j.0022-0337.2014.78.5.tb05728.x 24789836

[27] Anders PL , Davis EL , McCall WD Jr . Dental students’ glucometer experience and attitudes toward diabetes counseling, monitoring, and screening: a comparative study. J Dent Educ 2014;78(9):1263–7. 10.1002/j.0022-0337.2014.78.9.tb05797.x 25179922

[28] Gordon JS , Andrews JA , Albert DA , Crews KM , Payne TJ , Severson HH . Tobacco cessation via public dental clinics: results of a randomized trial. Am J Public Health 2010;100(7):1307–12. 10.2105/AJPH.2009.181214 20466951 PMC2882418

[29] National Center for Health Statistics. NHANES Response Rates and Population Totals. https://wwwn.cdc.gov/nchs/nhanes/ResponseRates.aspx#response-rates. Accessed June 3, 2020.

